# Connectivity mapping of glomerular proteins identifies dimethylaminoparthenolide as a new inhibitor of diabetic kidney disease

**DOI:** 10.1038/s41598-020-71950-7

**Published:** 2020-09-10

**Authors:** Julie Klein, Cécile Caubet, Mylène Camus, Manousos Makridakis, Colette Denis, Marion Gilet, Guylène Feuillet, Simon Rascalou, Eric Neau, Luc Garrigues, Olivier Thillaye du Boullay, Harald Mischak, Bernard Monsarrat, Odile Burlet-Schiltz, Antonia Vlahou, Jean Sébastien Saulnier-Blache, Jean-Loup Bascands, Joost P. Schanstra

**Affiliations:** 1 Institut National de la Santé et de la Recherche Médicale (INSERM), U1048, Institut of Cardiovascular and Metabolic Disease, Toulouse, France; 2 Université Toulouse III Paul-Sabatier, Toulouse, France; 3 Institut de Pharmacologie et Biologie Structurale (IPBS), UPS, CNRS, Université de Toulouse, Toulouse, France; 4 Biotechnology Laboratory, Centre of Basic Research, Biomedical Research Foundation of the Academy of Athens, Athens, Greece; 5 Laboratoire Hétérochimie Fondamentale et Appliquée, CNRS/Université Paul Sabatier, Toulouse, France; 6 Mosaiques Diagnostics GmbH, Hannover, Germany; 7 Institut National de la Santé et de la Recherche Médicale (INSERM), U1188 - Université de La Réunion, Saint-Denis, France; 8 Present Address: Evotec (France) SAS, Toulouse, France

**Keywords:** Proteomics, Kidney diseases, Diagnostic markers, Chronic kidney disease, Diabetic nephropathy, Computational biology and bioinformatics, Biomarkers, Nephrology, Drug discovery

## Abstract

While blocking the renin angiotensin aldosterone system (RAAS) has been the main therapeutic strategy to control diabetic kidney disease (DKD) for many years, 25–30% of diabetic patients still develop the disease. In the present work we adopted a systems biology strategy to analyze glomerular protein signatures to identify drugs with potential therapeutic properties in DKD acting through a RAAS-independent mechanism. Glomeruli were isolated from wild type and type 1 diabetic (Ins2Akita) mice treated or not with the angiotensin-converting enzyme inhibitor (ACEi) ramipril. Ramipril efficiently reduced the urinary albumin/creatine ratio (ACR) of Ins2Akita mice without modifying DKD-associated renal-injuries. Large scale quantitative proteomics was used to identify the DKD-associated glomerular proteins (DKD-GPs) that were ramipril-insensitive (RI-DKD-GPs). The raw data are publicly available via ProteomeXchange with identifier PXD018728. We then applied an in silico drug repurposing approach using a pattern-matching algorithm (Connectivity Mapping) to compare the RI-DKD-GPs’s signature with a collection of thousands of transcriptional signatures of bioactive compounds. The sesquiterpene lactone parthenolide was identified as one of the top compounds predicted to reverse the RI-DKD-GPs’s signature. Oral treatment of 2 months old Ins2Akita mice with dimethylaminoparthenolide (DMAPT, a water-soluble analogue of parthenolide) for two months at 10 mg/kg/d by gavage significantly reduced urinary ACR. However, in contrast to ramipril, DMAPT also significantly reduced glomerulosclerosis and tubulointerstitial fibrosis. Using a system biology approach, we identified DMAPT, as a compound with a potential add-on value to standard-of-care ACEi-treatment in DKD.

## Introduction

Diabetic kidney disease (DKD) has a high incidence (30–40%) in diabetic patients and about 50% of patients with DKD develop renal failure in the long term. In both human and animals, the presence of DKD is characterized by increased urinary albumin excretion resulting from apoptosis and functional loss of podocytes, resulting in impairment of glomerular filtration and glomerulosclerosis and, in more advanced DKD stages, tubulo-interstitial fibrosis^1,2^. Because glomerular injury marks the initial event of DKD, glomeruli are relevant targets to investigate the early molecular mechanisms of DKD pathogenesis.

The therapeutic options for preventing DKD are limited. Over the last decades, blockers of the renin angiotensin aldosterone system (RAAS, i.e. angio-converting enzyme inhibitors (ACEi) and angiotensin receptor blockers) have been the most widespread pharmacological treatments slowing down the development of DKD and its progression. However, 25–30% of the patients still develop DKD^3^.

Although RAAS inhibitors reduce DKD progression still 25–30% of the patients develop DKD^3^. Moreover, RAAS inhibitors can increase the risk of hyperkalemia when used at high doses or when combining two RAAS blockers^4^.

Therefore, additional therapies or drugs to prevent the progression of DKD are urgently needed. Emerging therapies undergoing clinical trials are focussing on expanding RAAS blockade with double angiotensin receptor/endothelin receptor blockers, antidiabetic drugs with additional nephroprotective properties such as sodium-glucose transporter 2 (SGLT2) inhibitors and glucagon-like peptide-1 (GLP-1) agonists, or targeting inflammation (pentoxifylline, a methylxanthine phosphodiesterase) or transcription factor Nrf2 (bardoxolone)^5^. Currently SGLT2 inhibitors appear to most promising news drugs in DKD treatment^6^. However, drugs used in these trials are focussing on single targets that will impact only part of the complex processes involved in the development of DKD. The complexity of DKD has prompted research to move away from investigating known candidate pathways and focusing on single molecules, to unbiased omics based studies^7^. This has resulted in a number of recent omics studies identifying potential biomarkers of DKD^8^, but not in novel drugs yet. This could be attributed to the fact that potential targets need to be transformed into drugs which often takes a substantial amount of time and effort. In addition, most studies employed genomic and transcriptomic strategies which do not necessarily reflect changes in proteins that are the major drug targets^9^.

In the present work we adopted a bioinformatic strategy of drug repurposing based on the analysis of protein signatures, aiming at finding new drugs that would complement the widely used RAAS blockers in DKD treatment. To this end, we established the glomerular protein signature of DKD in a mouse model of type I diabetes treated with ACEi versus control untreated animals; this signature was then compared with a database of thousands of molecular signatures of potential bioactive compounds (Connectivity MAP: https://portals.broadinstitute.org/cmap/). Connectivity Map (CMap) algorithms enable data-driven studies on drug repositioning^10,11^. Specifically it allows to compare a query gene signature to a differential gene expression database built by treating human cell lines with a wide range of chemical compounds. The analysis output is a list of compounds ranked according to their relevance to the query gene signature (positive or negative enrichment). CMap has been applied successfully in several studies including ours aiming at identifying candidate drugs for repurposing^12,13^. Here, we used the CMap resources and identified the sesquiterpene lactone parthenolide that was subsequently analyzed for its in vivo capacity to reduce the development of DKD in the type I diabetes mouse model.

## Results

### Influence of ramipril on the development of diabetic kidney disease

**Figure 1:**
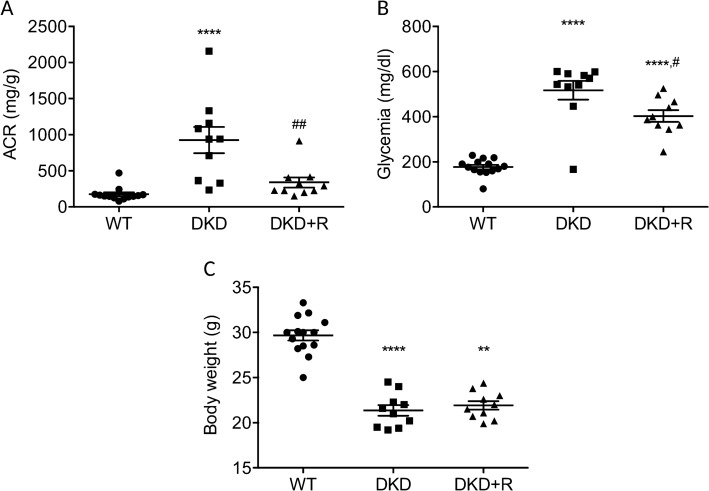
Influence of Ramipril-treatment on Ins2Akita mice. Urinary ACR (**A**), glycemia (**B**) and body weight (**C**) were measured in 4 months diabetic Ins2Akita that had been treated with (DKD + R) or without ramipril (DKD) for 2 months before sacrifice. Wild type (WT) mice of the same age were analyzed in parallel as a non-diabetic control. Values are mean ± SEM and One-way ANOVA test for multiple comparisons. Comparison with WT: **P < 0.01; ****P < 0.0001. Comparison between DKD and DKD + R: ^#^*P* < 0.05; ^##^*P* < 0.01.

Our investigations were performed in the Ins2Akita mouse since this model develops early to moderately advanced renal morphological changes and renal dysfunction as observed in type I DKD^14^. Moreover, ACEi reduces albuminuria in this model^15,16^. Ins2Akita mice (DKD) became significantly hyperglycemic at 1 month of age (Figure S3A) and exhibited significant increased ACR at 2 months (Figure S3B) compared to WT mice. Ramipril treatment, starting at 2 months of age, of Ins2Akita mice (DKD + R) significantly reduced ACR down to a level close to that of WT mice (Fig. 1A). These data confirmed the ability of ramipril to reduce albuminuria in Ins2Akita mice. Surprisingly, ramipril slightly (17%) but significantly (*p* = 0.038) reduced glycemia (Fig. 1B). This last observation was surprising because literature indicates that ramipril is not expected to influence (hyper)glycemia in a DKD mice^17,18^. Finally, ramipril had no influence on body weight (Fig. 1C).

### Identification of DKD-associated glomerular proteins insensitive to ramipril

Our objective was to identify DKD-associated glomerular protein (GP) that were unresponsive to ramipril. We hypothesized that these proteins could be potential targets for new pharmacological intervention in DKD on top of ACEi. To reach this objective, we aimed at identifying glomerular proteins (GPs) in four different groups of mice: WT, DKD, DKD + R and WT + R (8 mice per group). High quality glomeruli were isolated (see “Methods” section) from each mouse and GPs were identified and quantified by large-scale quantitative MS-based proteomics. Based on the analysis of the proteome of 32 glomerular samples, a total of 2,422 GPs were identified and quantified (Table S1).

#### Identification of the DKD-GPs

**Figure 2:**
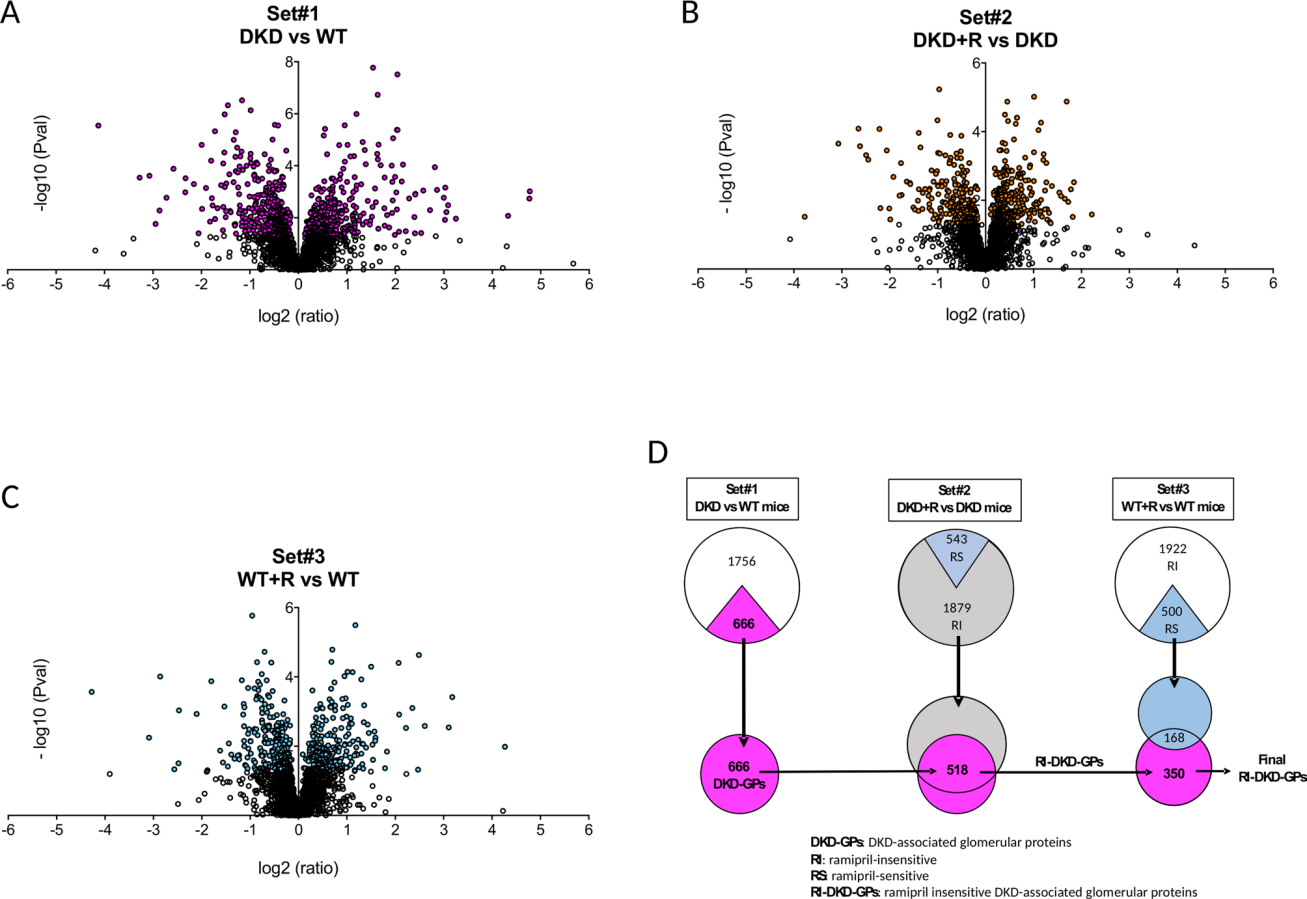
Selection of ramipril-insensitive DKD-associated glomerular proteins (RI-DKD-GPs). (**A**–**C**) Volcano-plot representation of the differential abundance of the glomerular proteins (GPs) in Set#1 (DKD vs WT) (**A**), Set#2 (DKD + R vs DKD) (**B**) and Set#3 (WT + R vs WT) (**C**) comparisons (colored circles: proteins representing a significant difference between the indicated comparison (*P* < 0.05); black circles: non-significant proteins). The Set#1 comparison led to the identification of 666 DKD-GPs out of which RI-DKD-GPs were selected according to their behavior in Set#2 and Set#3 comparisons. (**D**) Flowchart of RI-DKD-GPs selection (drawn using Microsoft PowerPoint for Mac, Version 16.16.19).

We first identified DKD-associated glomerular proteins (DKD-GPs) by comparing the abundance of GPs between DKD and WT mice (Set#1). This led to the identification of 666 GPs with significantly (*p* < 0.05) varying abundances (329 with increased and 337 with decreased abundance) (Fig. 2A and Table S1: columns AT-AV).

#### Identification of the RI-DKD-GPs

We then selected the DKD-GPs that were ramipril-insensitive (RI-DKD-GPs) according to their behavior when comparing DKD versus DKD + R mice (Set#2) and WT + R versus WT mice (Set#3) (identification flowchart in Fig. 2D). Set#2 (Fig. 2B) identified 1879 proteins with no significantly varying abundance. Among them, 518 proteins were also identified as DKD-GPs in Set#1 (DKD vs WT), and were classified as RI-DKD-GPs to indicate their insensitivity to ramipril treatment in a diabetic animals (Table S2: Filter 3). Nevertheless, 168 RI-DKD-GPs also showed significantly varying abundance in Set#3 (WT + R vs WT mice, Fig. 2C), indicating their sensitivity to ramipril-treatment in a non-diabetic animals (Table S2: Filter 4). These 168 proteins were therefore removed from the final RI-DKD-GPs list that now included 350 proteins (175 with increased 175 with decreased abundance, Table S2). RI-DKD-GPs represented 52% (350/666 * 100) of all DKD-GPs indicating ample space for improvement of DKD treatment.

### Identification of ramipril sensitive DKD-associated glomerular proteins

In parallel, we also isolated the DKD-GPs that were ramipril-sensitive (RS-DKD-GPs) (flowchart in Figure S4). Set#2 (DKD + R vs DKD) identified 543 proteins with significantly varying abundance. Among them, 86 had been identified as DKD-GPs in Set#1 (DKD vs WT) with an opposite trend of variation (Table S2: Filter 1). These 83 DKD-GPs were classified as RS-DKD-GPs to indicate their ability to be counter regulated by ramipril. Nevertheless, 3 of these 86 DKD-GPs behaved similarly (significant variation with opposite trend of variation than in Set#2) in Set#3 (WT + R vs WT) showing a sensitivity to ramipril not specific to DKD (Table S2: Filter 2). These 3 proteins were thus removed from the definitive RS-DKD-GPs list that finally included 83 proteins (35 up, 48 down) (Table S2).

### Pathway enrichment analysis of RI- and RS-DKD-GPs

Pathway analysis of RI-DKD-GPs showed a highly significant enrichment in proteins involved in the metabolism of amino acids, peroxisome, protein localization and fatty acid metabolism (Table 1). Pathway analysis of these RS-DKD-GPs showed a highly significant enrichment in proteins involved in small molecules transport and in folding of actin and tubulin by the CCT-TRIC complex (Table 2). These analysis suggested that RI-DKD-GPs were involved in quite different molecular pathways than RS-DKD-GPs, and may thus be potential targets for new pharmacological treatments of DKD.

**Table 1:** Top 10 significant pathways enriched in RI-DKD-GPs.

Gene set name	FDR q-value	Genes in overlap
Metabolism of amino acids and derivatives	7.19E−36	PIPOX, DAO, RPL18, RPL14, RPL11, RPS19, RPS7, RPS8, RPS17, RPL10A, RPL4, RPL7, RPL24, RPL28, RPL31, RPL32, Ah!IMP1, INMT, GLUL, PCBD1, PAPSS2, SCLY, PSMA2, PSMC4, PSMB2, PSME2, MCCC2, MCCC1, NQO1, GLUD1, GATM, GLS, ALDH4A1, ASS1, PRODH, HIBADH, PHGDH, GLDC, DMGDH, GCAT, GCSH, SLC25A15, HPD, HYKK, MPST, NAALAD2
Peroxisome	5.92E−23	PIPOX, DAO, ACAA1, HSD17B4, ECI2, CROT, HACL1, ACOX3, SCP2, EPHX2, NUDT19, PECR, AGPS, CAT, DHRS4, ABCD3, PEX3, PXMP4, FAR1, SOD2, NUDT12
Protein localization	3.27E−21	PIPOX, DAO, ACAA1, HSD17B4, ECI2, CROT, HACL1, ACOX3, SCP2, EPHX2, NUDT19, PECR, AGPS, CAT, DHRS4, ABCD3, PEX3, PXMP4, ACO2, SAMM50, FIS1, TOMM70, HSCB, TIMM10B, GFER
Fatty acid metabolism	5.96E−18	ACAA1, HSD17B4, ECI2, CROT, HACL1, ACOX3, SCP2, EPHX2, NUDT19, PECR, SLC22A5, ACAA2, HADHB, MCEE, ACSM3, PTGES2, GPX1, CYP4B1, ACOT12, ACOT1, ACSF2, ACAD11, PON3
Metabolism of lipids	5.96E−18	ACAA1, HSD17B4, ECI2, CROT, HACL1, ACOX3, SCP2, EPHX2, NUDT19, PECR, SLC22A5, ACAA2, HADHB, MCEE, ACSM3, PTGES2, GPX1, CYP4B1, ACOT12, ACOT1, ACSF2, ACAD11, PON3, AGPS, FAR1, ME1, MGLL, HSD17B11, GLB1, ARSB, RAB14, GM2A, RAN, CYP2D6, STS, SLCO1A2, OSBPL9, AKR1B15, FHL2, OSBPL5
Translation	2.23E−17	RPL18, RPL14, RPL11, RPS19, RPS7, RPS8, RPS17, RPL10A, RPL4, RPL7, RPL24, RPL28, RPL31, RPL32, AIMP1, EEF2, RPN1, SSR1, MRPL9, MRPS31, MRPL40, MRPS35, MRPS9, MRPS5, MRPL1, MRPS34, MRPL19
Genes encoding proteins involved in metabolism of fatty acids	1.22E−16	ACAA1, HSD17B4, ECI2, SLC22A5, ACAA2, HADHB, MCEE, ACSM3, ME1, MGLL, HSD17B11, ACO2, INMT, GLUL, PCBD1, ALDOA, UGDH, REEP6, RDH11, LGALS1, ERP29
Peroxisomal protein import	1.36E−15	ACAA1, HSD17B4, ECI2, CROT, HACL1, ACOX3, SCP2, EPHX2, NUDT19, PECR, AGPS, PIPOX, DAO, CAT, DHRS4
Selenoamino acid metabolism	2.71E−15	INMT, RPL18, RPL14, RPL11, RPS19, RPS7, RPS8, RPS17, RPL10A, RPL4, RPL7, RPL24, RPL28, RPL31, RPL32, AIMP1, PAPSS2, SCLY
Innate Immune System	2.58E−14	ACAA1, CAT, ALDOA, PTGES2, GLB1, ARSB, RAB14, GM2A, EEF2, PSMA2, PSMC4, PSMB2, PSME2, MAPK3, VCP, CAPZA1, CAPZA2, FYN, ARPC4, DNAJC3, GUSB, CTNNB1, PLCG2, FGB, CFL1, TKFC, CCT2, MLEC, NME2, HMGB1, CCT8, CTSH, PTPRJ, HPSE, GDI2, PDXK, S100A11, LYZ, DPP7, TMEM30A, SIGIRR, CFH, BPIFA2

Analysis was implemented with the GSEA software package (https://www.gsea-msigdb.org) with “Hallmarks gene sets” and “Canonical pathways” as Compute Overlaps.

**Table 2:** Top 10 significant pathways enriched in RS-DKD-GPs.

Gene set name	FDR q-value	Genes in overlap
Transport of small molecules	3.45E−8	PSMD7, OS9, AP2A2, PSMD12, CUBN, SLC4A1, PMPCB, ALB, ATP2A2, ATP2B2, ABCA9, SLC6A6, SLC9A1, SLC5A9, ATP11C
Folding of actin by CCT/TriC	3.55E−6	CCT4, CCT5, CCT3, CCT7
A subgroup of genes regulated by MYC-version 1 (v1)	5.53E−6	CCT4, CCT5, CCT3, CCT7, PSMD7, HSPD1, RACK1, SF3B3
Protein localization	1.94E−5	HSPD1, PMPCB, AMACR, ALDH3A2, DECR2, LDHD, ATAD1
Formation of tubulin folding intermediates by CCT/TriC	8.21E−5	CCT4, CCT5, CCT3, CCT7
Cooperation of Prefoldin and TriC/CCT in actin and tubulin folding	1.91E−4	CCT4, CCT5, CCT3, CCT7
Association of TriC/CCT with target proteins during biosynthesis	3.32E−4	CCT4, CCT5, CCT3, CCT7
Cooperation of PDCL (PhLP1) and TRiC/CCT in G-protein beta folding	4.00E−04	CCT4, CCT5, CCT3, CCT7
Asparagine N-linked glycosylation	6.35E−4	OS9, DYNC1H1, SPTAN1, DDOST, SPTBN1, DCTN5, MIA2
Neutrophil degranulation	1.14E−3	DYNC1H1, SPTAN1, DDOST, PSMD7, AP2A2, PSMD12, CD36, COMMD9

Analysis was implemented with the GSEA software package (https://www.gsea-msigdb.org ) with “Hallmarks gene sets” and “Canonical pathways” as Compute Overlaps.

### Connectivity mapping of RI-DKD-GPs signature

In an attempt to identify a pharmacological compound able to inhibit DKD through a ramipril-independent mechanism, RI-DKD-GPs were analyzed in silico using both CMap1 and CMap2 as recommended by Lim and Pavlidis^19^ (“Methods” section). Using CMap1, we found 2 top compounds (quizapine and parthenolide) that exhibited the highest negative enrichment score with best “percent non-nul” (100) (Table 3). Quizapine is a serotonin receptor agonist. Parthenolide is a sesquiterpene lactone naturally present in a plant (*Tanacetum parthenium*)^20^. When using CMap2 (Table 4), quizapine was not retrieved, but parthenolide was found twice within the top 20 compounds with highest negative enrichment as two different diastereoisomers (BRD IDs K28120222 and K98548675 at the 10th and 16th row respectively). BRD ID, is a compound identifier developed by the Broad Institute (https://clue.io/connectopedia/what_is_a_brd_id). These observations suggested that parthenolide could have the potential to impact on the ramipril-insensitive glomerular DKD protein signature and therefore the DKD phenotype.

**Table 3:** Top 20 compounds predicted to reverse RI-DKD-GPs protein signature following CMap 1 analysis.

CMap name	Mean	n	Enrichment	p	Specificity	Percent non-null	Description
Quipazine	− 0.585	4	− 0.88	0.00052	0	100	Serotonin receptor agonist
**Parthenolide**	− 0.738	4	− 0.872	0.00056	0.0138	100	NFkB pathway inhibitor
Cephaeline	0	5	− 0.809	–	–	0	Protein synthesis inhibitor
Suxibuzone	− 0.453	4	− 0.79	0.00402	0	75	Anti-inflammatory
Flavoxate	− 0.179	4	− 0.736	–	–	25	Acetylcholine receptor antagonist
Mephenesin	− 0.137	5	− 0.728	–	–	20	Myorelaxant
Amphotericin B	− 0.318	4	− 0.701	0.01649	0.0385	50	Antifungi
Sulfamethizole	− 0.241	4	− 0.698	0.01745	0.017	50	Antibiotic
Ceforanide	− 0.526	4	− 0.691	0.01959	0.024	75	Penicillin binding protein inhibitor
Ifenprodil	0	4	− 0.687	–	–	0	Adrenergic receptor antagonist
Zoxazolamine	− 0.149	4	− 0.686	–	–	25	Myorelaxant
Pyridoxine	0	4	− 0.686	–	–	0	Vitamin B6
Antazoline	− 0.317	4	− 0.67	0.02668	0.0315	50	Histamine receptor antagonist
Puromycin	− 0.154	4	− 0.657	–	–	25	Protein synthesis inhibitor
Cisapride	− 0.147	4	− 0.646	–	–	25	Serotonin receptor agonist
Nifenazone	− 0.364	5	− 0.64	0.01544	0.0132	60	Analgesic
Emetine	0	4	− 0.633	–	–	0	Protein synthesis inhibitor
Cinchonine	− 0.427	4	− 0.631	0.04538	0.1975	75	P-glycoprotein inhibitor
Piperacetazine	− 0.283	4	− 0.629	0.04629	0.0438	50	Dopamine receptor antagonist
Natamycin	0	4	− 0.628	–	–	0	Antibiotic

The classification is based on the score of negative enrichment (the closer to − 1 the better) of the small molecules with a n ≥ 4. Abbreviations: “Mean”, is the arithmetic mean of the connectivity scores for those signatures; “n” is the number of signatures of a given compound available in the Cmap database; ”enrichment” indicates the degree of matching between a query signature (here the RI-DKD-GPs signature) and the reversed signature of a given compound; “p” (permutation p) estimates the likelihood that the enrichment would be observed by chance; “specificity” provides a measure of the uniqueness of the matching between a compound and the CVD signature; “percent non-nul” measures the support for the connection between a set of compound signature and the query signature based upon the behavior of the individual signature in that set. More details can be found here: https://portals.broadinstitute.org/cmap/help_topics_frames.jsp.

**Table 4:** Top 20 compounds predicted to reverse RI-DKD-GPs protein signature following CMap 2 analysis.

Score	Name	Description
− 96.05	MLN-2238	Proteasome inhibitor
− 94.25	NSC-632839	Ubiquitin specific protease inhibitor
− 92.95	Linifanib	PDGFR receptor inhibitor
− 92.82	Droxinostat	HDAC inhibitor
− 92.18	MG-132	Proteasome inhibitor
− 91.66	z-leu3-VS	Proteasome inhibitor
− 91.01	Securinine	GABA receptor antagonist
− 90.99	BNTX	Opioid receptor antagonist
− 89.94	BCI-hydrochloride	Protein phosphatase inhibitor
− 89.15	**Parthenolide**	NFkB pathway inhibitor
− 88.73	Thiostrepton	FOXM1 inhibitor
− 88.15	BMY-45778	IP1 prostacyclin receptor agonist
− 87.23	Piperlongumine	Glutathione transferase inhibitor
− 85.58	Radicicol	HSP inhibitor
− 85.57	Amonafide	Topoisomerase inhibitor
− 84	**Parthenolide**	NFkB pathway inhibitor
− 83.93	BI-2536	PLK inhibitor
− 83.76	BAY-K8644	Calcium channel activator
− 82.28	Ofloxacin	Bacterial DNA gyrase inhibitor
− 80.96	Devazepide	CCK receptor antagonist

The Score is a standardized measure ranging from − 100 to 100 corresponds to the fraction of reference gene sets with a greater similarity to the perturbagen than the current query. More details can be found here: https://clue.io/connectopedia/connectivity_scores.

### Influence of parthenolide on DKD

Following the CMap prediction, the capacity of parthenolide to inhibit DKD was further investigated. Parthenolide has a poor water-solubility, constituting a major limitation for in vivo studies and further development as a clinical therapeutic agent. To address this issue, an orally bioavailable analog of parthenolide, DMAPT (dimethyamino-parthenolide, fumarate salt) was developped^21,22^. We synthesized DMAPT (see Supplementary materials) and its impact was tested on the development of DKD in Ins2Akita mice and was compared to ramipril.

**Figure 3:**
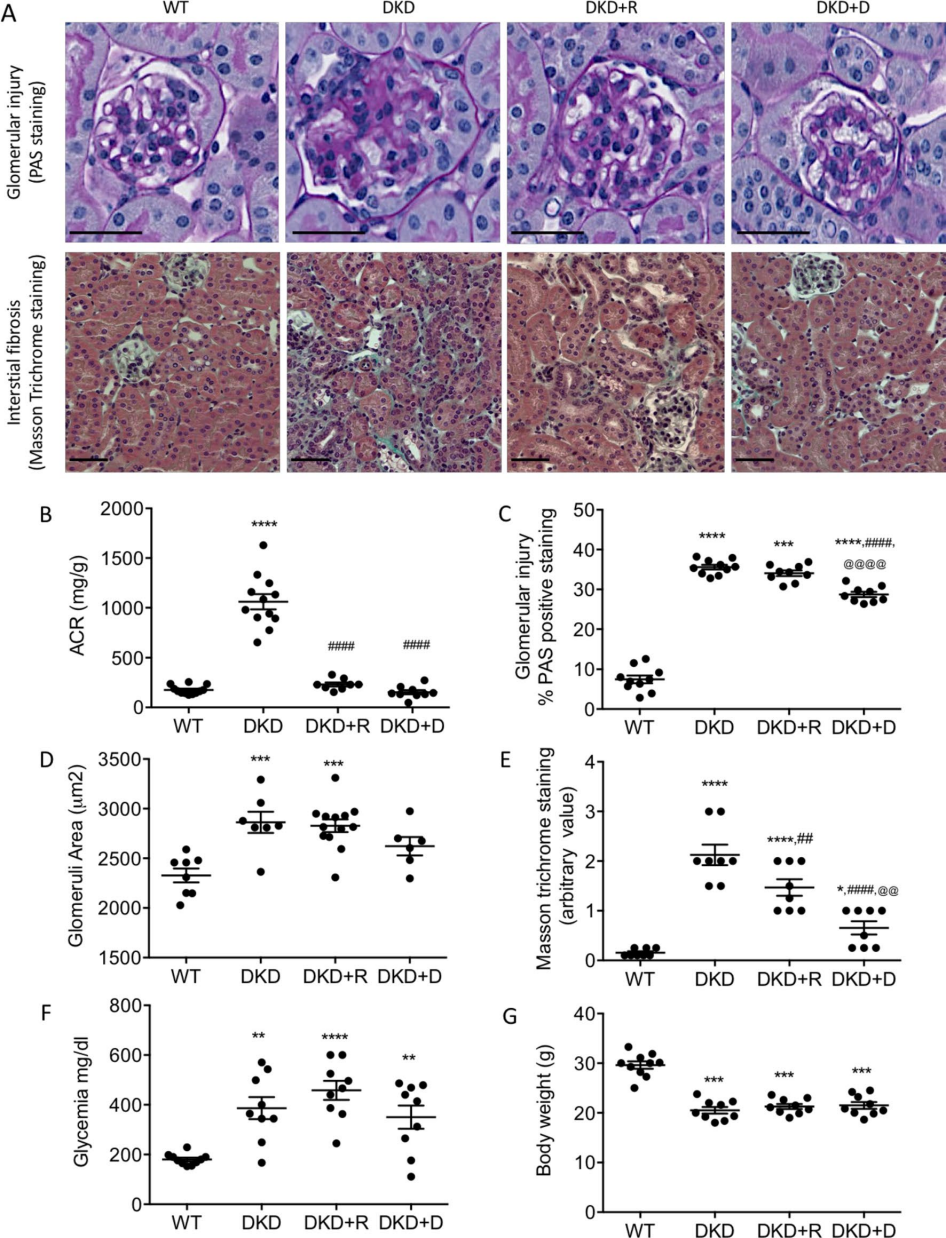
Comparative influence of DMAPT- and Ramipril-treatment on Ins2Akita mice. (**A**) representative glomerular injury (PAS staining) and interstitial fibrosis (Masson trichrome) in kidneys from 4 month old diabetic Ins2Akita (DKD) that had been treated or not with Ramipril (DKD + R) or DMAPT (DKD + D) for 2 months before sacrifice (scale bar = 50 µm). (**B**–**G**) Quantification of ACR (**B**), glomerular injury (**C**), glomerular area (**D**), fibrosis (**E**), glycemia (**F**), and body weight (**G**) in WT (n = 10), DKD (n = 9), DKD + R (n = 9) and DKD + D (n = 9). Values are mean ± SEM and One-way ANOVA test for multiple comparisons. Comparison to wild type mice (WT): *P < 0.05; **P < 0.01; ***P < 0.001; ****P < 0.0001. Comparison of DKD + R or DKD + D to DKD: ^#^*P* < 0.05; ^##^*P* < 0.01; ^###^*P* < 0.001; ^####^*P* < 0.0001. Comparison of DKD + R to DKD + D: ^@@^*P* < 0.01; ^@@@@^*P* < 0.0001.

Treatment of Ins2Akita mice (DKD) with DMAPT (DKD + P) led to a significant reduction of ACR to the same extent to that of ramipril (DKD + R) (Fig. 3B). DMAPT also significantly reduced glomerular injury (PAS staining) (Fig. 3A,C) and fibrosis (Masson trichrome staining) (Fig. 3A,E), and tended to reduce glomerular area, although without reaching significance (Fig. 3A,D). In contrast, ramipril had no significant influence on these 3 parameters (Fig. 3A–E). Neither glycemia (Fig. 3F) nor body weight (Fig. 3G) were significantly influenced by DMAPT and ramipril. The absence of effect of ramipril on glycemia contrasted with the slight reduction that was observed in the first experiment (Fig. 1B) but was in line with what was previously reported^17,18^. In conclusion, these data indicated that DMAPT inhibits DKD-associated albuminuria and that, in contrast to ramipril, DMAPT also attenuates DKD-associated kidney injuries.

## Discussion

At least in part as a result of their antihypertensive activity, ACEi have been one of the most widespread treatment of DKD progression but 25–30% of the patients still develop DKD^3^. Therefore, additional therapies or drugs complementing ACEi to prevent the progression of DKD are urgently needed.

Here, we found that among the glomerular proteins that are modified during DKD in Ins2Akita mouse, a model of moderately advanced type I DKD, only a small proportion (12%) is affected (counter regulated) by the ACEi ramipril. The remaining proteins apparently insensitive to ramipril may represent potential targets for new drug-treatment of DKD through a ramipril independent mechanism. By browsing the “drug repurposing” data base CMap, we found that the best negative enrichment of the ramipril-insensitive glomerular DKD protein signature is obtained with parthenolide, predicting that this molecule could potentially inhibit DKD progression. Since CMap analysis is based on transcriptome data, we had to convert the protein IDs into gene IDs. This constitutes a limitation of applying the CMap algorithms to proteome data because a protein profile is not necessarily correlated with a mRNA profile. Unfortunately, to the best of our knowledge, there currently does exist a drug repurposing algorithm applicable to proteomics data.

Parthenolide is a sesquiterpene lactone naturally present in a plant (Tanacetum parthenium), with anti-cancer and anti-inflammatory effects^23^. Parthenolide was shown to have a beneficial impact on proteinuria and renal injury in immune glomerulonephritis in rat^24^, but to the best of our knowledge the beneficial impact of parthenolide on DKD has not been reported.

Parthenolide has a poor water-solubility that limits its clinical use. Here we show that treatment with an orally bioavailable analog of parthenolide, DMAPT, potently reduces urinary ACR in Ins2Akita mice, verifying in vivo the in silico prediction with CMap. Moreover, we showed that DMAPT is also able to reduce kidney lesions associated with DKD in Ins2Akita mice. This is contrasting with the absence of effects of ramipril-treatment on kidney lesions seen in our and other studies^15,16^. In addition, to our knowledge, this is the first demonstration of the in vivo use of orally administered DMAPT to treat DKD.

The biological effects of DMAPT and parthenolide are mediated at least in part via inhibiting the activity of the NF kappa B transcription factor complex^20^. Therefore, the beneficial impact of DMAPT on kidney injuries could partially depend on the NF kappa B dependent pathways. The hypothesis is in agreement with previous reports showing that dysregulation of NF kappa B is involved in podocyte damage and proteinuria in DKD^25–29^. The hypothesis is also in agreement with a previous report showing that parthenolide is also able to alleviate renal inflammation and insulin resistance in type 2 diabetic db/db mice^30^. This is in agreement with the fact that NF kappa B plays a key role in immune response, and suggests that the beneficial effects of DMAPT could also be mediated by inhibiting immune and inflammatory cells. Single cell profiling methods, such as single cell RNA-seq or single cell westerns could be conducted to test such hypothesis. In addition, DMAPT and parthenolide were also reported to inhibit histone deacetylase (HDAC) activity and this effect is independent of NF kappa B^31,32^. In fact, several reports support the renoprotective effects of HDAC inhibitors in experimental DKD^33^. Therefore, the protective effect of DMAPT and parthenolide in DKD could be the result of affecting multiple disease-relevant pathways.

Although further studies are required to test the add-on effect of DMAPT in DKD (in both type I and II diabetes), our data strongly suggested that parthenolide or its derivatives may be potential new drug candidates for DKD treatment, advantageously complementing ACEi. A phase I trial with standardized doses in patients with cancer showed that parthenolide was well tolerated without dose-limiting toxicity^34^. However, no data on potential renoprotective effects were presented in this report. Whether parthenolide could be used in patients with DKD remains to be tested.

## Methods

### Animals

Male C57BL/6-*Ins2*^*Akita*^/J (Ins2Akita) and C57BL/6J (WT) mice (Charles River) were housed with unrestricted access to water and were maintained on a 12-h light–dark cycle in a pathogen-free environment on standard mouse chow. All experiments were conducted in accordance with the Guide for the care and use of laboratory animals of the National Institute of Health, eighth edition and the French Institute of Health guidelines for the care and use of laboratory animals. The project was approved by the local (Inserm/UPS US006 CREFRE) and national ethics committees (ethics committee for animal experiment, CEEA122; Toulouse, France; approval 02867.01).

### Treatments and urine collection

In a first series of experiments, 2 months old WT and Ins2Akita mice were treated with or without ACEi ramipril (10 mg/kg/d in drinking water) for 2 months. In a second series of experiments 2 months old WT and Ins2Akita mice were treated with or without ACEi ramipril as in the first series or with dimethylaminoparthenolide monofumarate (DMAPT, 10 mg/kg/d by gavage) for 2 months. In each series of experiments, urine was collected in metabolic cages for 12 h, few days before sacrifice.

### Isolation of glomeruli

Glomeruli were isolated as previously described^35^ with minor modifications. Mice were anesthetized by ip injection of a mixture of ketamine (100 mg/kg) and xylazine (10 mg/kg) in phosphate buffered saline (PBS). A catheter was placed into the abdominal aorta after ligation of the cava vein, the upper abdominal aorta, the mesenteric and the celiac arteries. The lower part of the abdominal aorta was perfused with 40 ml of Dynabeads M-450 Tosylactivated (4.5 µm diameter, Dynal A.S., Oslo, Norway) at a concentration of 2 × 10^6^ beads/ml followed by 15 ml of cold PBS. This procedures allows accumulation of beads in glomeruli^36^. Next, the left and right kidneys were collected and decapsulated. The animal was sacrificed by cervical dislocation and blood was collected by intra-cardiac puncture in heparinized tubes and plasma was prepared by centrifugation at 1500×*g* at 4 °C for 5 min and stored at − 20 °C for further use. One portion of the right kidney was snap-frozen in liquid nitrogen and stored at − 80 °C. Another portion of the right kidney cortex was fixed in Carnoy’s solution (ethanol/chloroform/glacial acetic acid: 60/ 30/10, v/v/v) for further histological analysis.

The left kidney was gently pressed manually through a 70 µm cell strainer using a flattened pestle followed by washing of the cell strainer with 20 ml of cold PBS. The filtrate was centrifuged at 200×*g* for 5 min at 4 °C, and the glomerular pellet was adjusted to 2 ml with PBS and transferred to an Eppendorf tube that was placed in a magnetic particle concentrator (Dynal A.S., Oslo, Norway) to concentrate the glomeruli into a pellet. The supernatant was discarded and the pellet was washed 5 × with 1 ml of PBS. The final pellet was resuspended in 100 µl PBS. This procedure allows the isolation of ~ 4,000 glomeruli per kidney.

Based on light microscopy survey, our glomerular suspensions were highly enriched for glomeruli (Figure S2A,B). Moreover, mRNA quantification showed that isolated glomeruli were enriched in glomerular-specific genes (nphs2, podxl and cldn5) while proximal (aqp1, slc22a13), distal tubules (wnk), and of loop of Henle (aqp1, slc12a3) specific genes were not, or poorly enriched compared to total kidney (Figure S2). Moreover, among the 2,422 proteins detected by LC-MS/MS, several glomerular-specific proteins were ranked within the first upper quartile of relative abundance (columns AR-AS, Table S1) including NphS1 (rank 196), Tjp1/ZO-1 (rank 221) Nphs2 (rank 339), Synpo (rank 388), Actn4 (rank 7), cd2ap (rank 543), while several tubular-specific proteins Aqp1 (rank 591), Umod (rank 1634) were ranked as less abundant proteins, or were not detected (slc22a13, Wnk). These observations support the relative high purity of our glomerular preparations.

### Renal histology

Right kidney cortexes were fixed in Carnoy’s solution for 24 h, dehydrated in successive baths with increasing alcohol concentrations, embedded in paraffin and 2 µm sections were cut and mounted, and stained with either periodic acid-Schiff (PAS) or Masson trichrome. Sections were digitalized with a Nanozoomer 2.0 RS (Hamamatsu Photonics SARL) and glomerular surface was quantified with Morpho-expert software (version 1.00, Explora Nova). At least 50 glomeruli including superficial and juxtaglomerular cortical area, were examined for each animal. The extent of glomerular injury was expressed as the percentage of glomerular area fraction occupied by PAS positive matrix^37^.

### Biochemical analysis

Urinary albumin concentration was measured by ELISA using the AlbuWell kit (WAK-Chemie Medical GmbH, Steinbach, Germany). Urinary creatinine concentration was measured by the colorimetric method of Jaffe according to the protocol Creatinine Assay Kit (Bio Assay Systems). Blood glucose levels were measured in caudal blood from fasted awake mice using a glucometer (Glucometer Elite XL; Bayer Healthcare, Elkhart, IN).

### Quantitative proteomics of glomerular samples

#### Glomerular sample preparation for proteomics

Isolated glomeruli were homogenized in RIPA buffer under agitation for 3 min and centrifuged 15 min at 13,000×*g* to pellet the beads together with cell debris. The supernatants were collected and stored at − 80 °C at a protein concentration of 1–2 mg/ml before being processed for mass spectrometry (MS) analysis^38^. Protein samples were air-dried using a SpeedVac concentrator and reconstituted in 1 × final Laemmli buffer containing dithiothreitol (25 mM) and heated at 95 °C for 5 min. Cysteines were alkylated in chloroacetamide (75 mM) for 30 min at room temperature. Proteins (10 µg) were loaded onto a 12% acrylamide SDS-PAGE gel and concentrated in a single band visualized by Coomassie staining (Instant Blue—Expedeon). The gel band containing the whole sample was cut and washed in 50 mM ammonium bicarbonate:acetonitrile (1:1) for 15 min at 37 °C. Proteins were in-gel digested using 0.6 μg of modified sequencing-grade trypsin (Promega) in 50 mM ammonium bicarbonate overnight at 37 °C. Peptides were extracted from the gel by two incubations in 10% formic acid:acetonitrile (1:1) for 15 min at 37 °C. Extracted fractions were pooled with the initial digestion supernatant and dried under speed-vaccum. The resulting peptides were resuspended with 14 µL of 5% acetonitrile, 0.05% trifluoroacetic acid for nanoLC-MS/MS analysis.

#### NanoLC-MS/MS analysis

Peptides were analyzed by nanoLC-MS/MS using an UltiMate 3000 system (Dionex) coupled to an LTQ Orbitrap Velos ETD mass spectrometer (Thermo Fisher Scientific)^38^. Each sample (5 µl) was loaded onto a C18 precolumn (300 μm inner diameter × 5 mm; 5 µm particule size; 100 Å pore size; Dionex) at 20 μl/min in 5% acetonitrile, 0.05% trifluoroacetic acid. After 5 min of desalting, the precolumn was switched online with the analytical C18 column (75 μm inner diameter × 50 cm; 3 µm particule size; 120 Å pore size in-house; packed with Reprosil C18) and was equilibrated in 95% solvent A (5% acetonitrile, 0.2% formic acid) and 5% solvent B (80% acetonitrile, 0.2% formic acid). Peptides were eluted using a 5–50% gradient of solvent B over 110 min at a flow rate of 300 nl/min. The mass spectrometer was operated in a data-dependent acquisition mode with Xcalibur software. Survey MS scans were acquired in the Orbitrap on the 300–2000 *m/z* range with the resolution set at 60,000. The 20 most intense ions per survey scan were selected for CID fragmentation and the resulting fragments were analyzed in the linear ion trap (LTQ). A dynamic exclusion of 60 s was used to prevent repetitive selection of the same peptide. Each sample was injected once for MS analysis.

#### Protein identification and quantification from raw nanoLC-MS/MS data

Raw nanoLC-MS/MS files were processed with the MaxQuant software (version 1.5.2.8) for database search with the Andromeda search engine and for quantitative analysis^39^. Data were searched against “*Mus musculus*” entries in the Swiss-Prot protein database (UniProtKB/Swiss-Prot protein knowledgebase release 2013_06; 16,641 sequence entries of Mus musculus). Carbamidomethylation of cysteine was set as a fixed modification whereas oxidation of methionine and protein N-terminal acetylation were set as variable modifications. Specificity of trypsin digestion was set for cleavage after K or R and two missed trypsin cleavage sites were allowed. The precursor mass tolerance was set to 20 ppm for the first search and 4.5 ppm for the main Andromeda database search. The mass tolerance in MS/MS mode was set to 0.8 Da. Minimum peptide length was set to 7 amino acids and minimum number of unique peptides was set to 1. Andromeda results were validated by the target-decoy approach using a reverse database at both a peptide and protein FDR of 1%. For label-free relative quantification of the samples, the “match between runs” option of MaxQuant was enabled with a time window of 3 min to cross-assign the MS features detected in the different runs.

#### Data processing and statistical analysis

Protein entries identified as potential contaminants from the ‘proteinGroups.txt’ files generated by MaxQuant were eliminated from the analysis, as were proteins identified by fewer than two peptides. Protein relative quantification was performed by comparisons of different groups of eight samples each (8 biological replicates per group: WT, DKD, DKD + R, WT + R) (Table S1). Protein intensities were normalized across all conditions by the median intensity. For each comparison, only proteins which were quantified in at least 4 biological replicates (4 intensities values retrieved by MaxQuant) in at least one of the groups were considered for further processing and statistical analysis (Filter 1, columns AR to AU, Table S1). Remaining missing values were then replaced by a constant noise value determined independently for each analytical run as the 1% percentile of the total protein population. The mean proportion of missing values over the whole analytic run was 2.1% (line 2,435, column S, Table S1). Proteins with a *p* value of less than 0.05 (T-test) were considered as significantly varying between two groups.

#### Proteomic data availability

The mass spectrometry proteomics raw data have been deposited to the ProteomeXchange Consortium via the PRIDE^40^ partner repository with the dataset identifier PXD018728.

### Pathway enrichment analysis

Pathway enrichment analysis of up- and down-regulated proteins was using the Gene Set Enrichement Analysis (GSEA) software package from the Molecular Signatures Database (MSigDB) (https://www.gsea-msigdb.org)^10,41–43^ using “Hallmarks gene sets” and “Canonical Pathways” as Compute Overlaps. MSigDB is a joint project of UC San Diego and Broad Institute.

### Connectivity Map analysis

The initial version of CMap (CMap1: https://portals.broadinstitute.org/cmap) consists of 6,100 differential expression profiles obtained after treatment of 3 cultured human cells (MCF7, PC3, and HL60) with varying concentrations of 1,309 compounds^11^. More recently, a new CMap version was released^44^ (CMap2: https://clue.io/) encompassing 8,549 differential expression profiles obtained after treatment of 9 cultured human cells (VCAP, A375, A549, HAE1, HCC515, HEPG2, HT29, MCF7, PC3) with varying concentrations of 2,428 compounds. For our experiments, each mouse protein ID was first converted to its human ortholog and then converted into human gene ID. Up- and down-gene IDs were then queried to CMap1 and CMap2 to retrieve compounds with best negative enrichment as recently recommended^19^.

### DMAPT synthesis

Dimethylaminoparthenolide monofumarate [(13-(*N*,*N*-dimethyl)-amino-4a,5b-epoxy-4,10-dimethyl-6a-hydroxy-12-oic acid-c-lactonegermacra-1(10)-ene monofumarate)] was synthesized by reaction of parthenolide (Sigma-Aldrich) with dimethylamine (Sigma-Aldrich) and isolated as the fumarate salt as previously described^21^. Analytical data (^1^H and ^13^C NMR, mass spectrometry and melting point) are consistent to those previously reported^16^. DMAPT fumarate purity was checked by elemental analysis and was evaluated > 98% (Supplementary Methods and Figure S1).

### Statistics

Comparison between 2 groups of values was implemented using a two-tailed unpaired Welch’s t-tests. Comparison between more than 2 groups, was implemented using an ordinary one-way ANOVA followed by Homl–Sidak’s multiple comparisons test was used. *P* < 0.05 was considered statistically significant. For the proteomic data the *P* values were adjusted for the false discovery rate (Benjamini–Hochberg).

## Supplementary information

Supplementary Figures.

Supplementary Table S1.

Supplementary Table S2.

Supplementary Legends.
