# Supplementary material for: Connectivity mapping of glomerular proteins identifies dimethylaminoparthenolide as a new inhibitor of diabetic kidney disease
Source: Sci Rep. 2020 Sep 10;10:14898. doi: 10.1038/s41598-020-71950-7 (PMC7484761; doi:10.1038/s41598-020-71950-7)
Supplement: Supplementary file 1 — Supplementary Figures. [file 41598_2020_71950_MOESM1_ESM.pdf]

Connectivity mapping of glomerular proteins identifies dimethylaminoparthenolide as a new inhibitor of diabetic kidney disease.

Julie Klein[1,2], Cécile Caubet[1,2,γ], Mylène Camus[3], Manousos Makridakis[4], Colette Denis[1,2], Marion Gilet[1,2], Guylène Feuillet[1,2], Simon Rascalou[1,2], Eric Neau[1,2], Luc Garrigues[3,γ], Olivier Thillaye du Boullay[5], Harald Mischak[6], Bernard Monsarrat[3], Odile Burlet-Schiltz[3], Antonia Vlahou[4], Jean Sébastien Saulnier-Blache[1,2,*], Jean-Loup Bascands[7,*], Joost P. Schanstra [1,2,*].

[1] Institut National de la Santé et de la Recherche Médicale (INSERM), U1048, Institut of Cardiovascular and Metabolic Disease, Toulouse, France.
[2]Université Toulouse III Paul-Sabatier, Toulouse, France.
[3] Institut de Pharmacologie et Biologie Structurale (IPBS), Université de Toulouse, UPS, CNRS, Toulouse, France.
[4]Biotechnology Laboratory, Centre of Basic Research, Biomedical Research Foundation of the Academy of Athens, Athens, Greece.
[5] Laboratoire Hétérochimie Fondamentale et Appliquée, CNRS/Université Paul Sabatier, Toulouse, France.
[6] Mosaiques Diagnostics GmbH, Hannover, Germany.
[7] Institut National de la Santé et de la Recherche Médicale (INSERM), U1188 - Université de La Réunion, France.

Supplementary Methods and Results.

Chemical synthesis of Dimethylaminoparthenolide monofumarate.

Dimethylaminoparthenolide monofumarate [(13-(N,N-dimethyl)-amino-4a,5b-epoxy-4,10-dimethyl-6a-hydroxy-12-oic acid-c-lactonegermacra-
1(10)-ene monofumarate)] was synthesized by reaction of parthenolide (Sigma-Aldrich) with dimethylamine (Sigma-Aldrich) and isolated as the fumarate salt as previously described (*Neelakantan and al. Bioorg. Med. Chem. Lett.* ***19****, 4346–4349, 2009*).

The NMR spectra were recorded on Bruker Avance II 300 MHz apparatus at 298K. ^{1}H and ^{13}C chemical shifts reported are referenced internally to residual protio-(1H) or deutero-(13C) solvent. Chemical shifts (δ) are expressed in parts per million (ppm).
Mass spectrometry (MS) spectra were measured with a Hewlett-Packard 5989A in the electron impact mode (70 eV). High-resolution mass spectrometry (HRMS) spectra were measured with a GCT Premier Waters in DCI mode (CH_4).
Melting points were measured in a sealed capillary using the Stuart automatic melting point SMP40 apparatus.
Elemental Analyses (microanalyses C, H, N) were performed by the Laboratoire de chimie de coordination (LCC, Toulouse) on a PerkinElmer 2400 Series II system.

13-(N,N-dimethyl)-amino-4a,5b-epoxy-4,10-dimethyl-6a-hydroxy-12-oic acid-c-lactone-germacra-1(10)-ene monofumarate (DMAPT monofumarate):

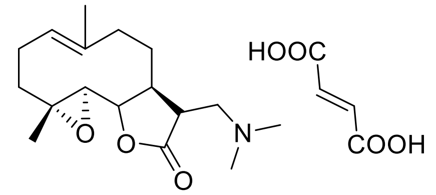

DMAPT synthesis - To a solution of parthenolide (1.40 g, 5.64 mmoles) in methanol (140 mL) was added at room temperature and under argon a solution of dimethylamine in methanol (2.7 M, 7 mL, 18.9 mmoles). The reaction mixture was stirred at room temperature (16h) until full consumption of parthenolide (TLC, DCM 95/ MeOH 5). Excess of dimethylamine was removed by flushing the reaction mixture with argon. The solvent was removed under reduced pressure and the crude was purified by flash chromatography (combiflash, 40 g of silica, DCM/MeOH (gradient 1 to 5% methanol) to yield a white-off powder (1.56 g, 94%).

^{1}H NMR (300 MHz, $CDCl_3$): δ 5.18 (1H, dd,), 3.82 (1H, t), 3.73 (2H, dd), 2.62 (1H, dd), 2.01 – 2.50 (8H, m), 2.26 (6H, s), 1.55 (1H, m), 1.69 (3H, s), 1.29 (3H, s), 1.22 (1H, m) ppm. (**Figure S1A**)

^{13}C NMR (75 MHz, $CDCl_3$): δ 176.5, 134.7, 125.1, 82.1, 66.5, 61.5, 57.7, 47.9, 46.5, 46.3, 41.1, 36.7, 30.0, 24.1, 17.3, 17.0 ppm. (**Figure S1B**)

MS (APCI) : 294.40

DMAPT fumarate synthesis - A solution of fumaric acid (620 mg, 1 equivalent) in a mixture of DCM (30 mL) and EtOH (20 mL) was added to a solution of free DMAPT (1.56 g, 5.3 mmoles) in DCM (200 mL). The mixture was stirred overnight under argon atmosphere. The resulting salt was filtered-off and washed with DCM and dried under vacuum to yield a white powder (1.95 g, 89%).

Melting point : 184–186 °C

^{1}H NMR (300 MHz, D_2O): δ 6.60 (2H, s), 5.18 (1H, dd), 4.24 (1H, t), 3.54 (1H, dd), 3.36 (1H, dd), 3.13 (1H, td), 3.01 (1H, d), 2.91 (6H, s), 1.80–2.45 (8H, m), 1.63 (3H, s), 1.27 (3H, s), 1.15 (1H, td) ppm. (**Figure S1C**)

^{13}C NMR (75 MHz, D_2O): δ 177.4, 135.8, 134.7, 124.9, 83.3, 66.6, 64.9, 55.7, 47.1, 42.3, 40.0, 35.5, 28.3, 23.5, 16.1, 15.9 ppm. (**Figure S1D**)

Elemental Analysis $C_{21}H_{31}NO_7$ (DMAPT monofumarate)
Calculated: C : 61,60; H : 7,63; N : 3,42.
Experimental: C : 61,62; H : 7,96; N : 3,40.

HRMS (ESI) Calculated for $[M+H^+]$ ($C_{17}H_{28}NO3$): 294,2069
Experimental : 294,2071

Figure S1 A, B, C, D: Analytical data (^{1}H and ^{13}C NMR, mass spectrometry and melting point) are consistent to those previously reported (*Neelakantan and al. Bioorg. Med. Chem. Lett.* ***19,*** *4346–4349, 2009*). DMAPT fumarate purity was checked by elemental analysis.

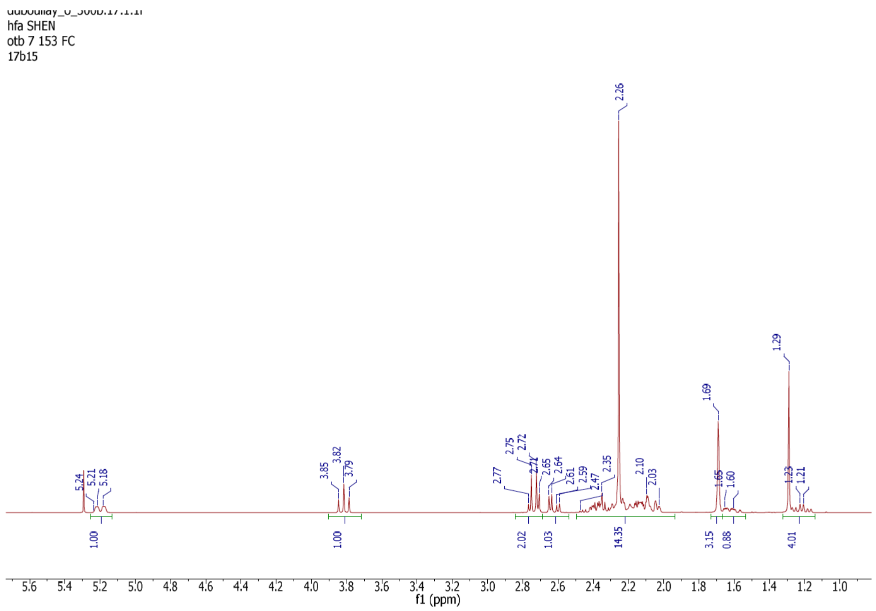

Figure S1A : DMAPT, ^{1}H NMR (300MHz, $CDCl_3$).

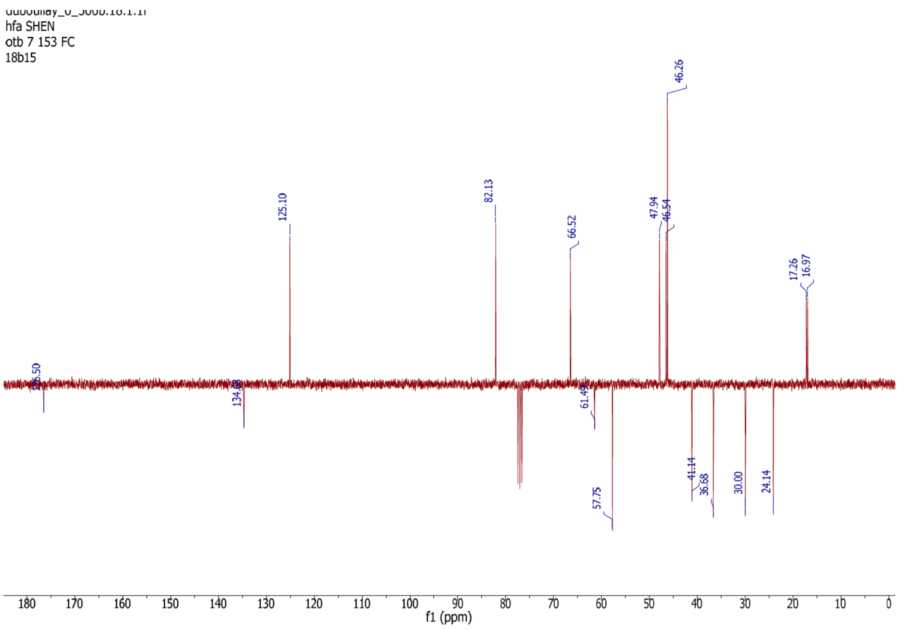

Figure S1B : DMAPT, ^{13}C NMR (75 MHz, $CDCl_3$)

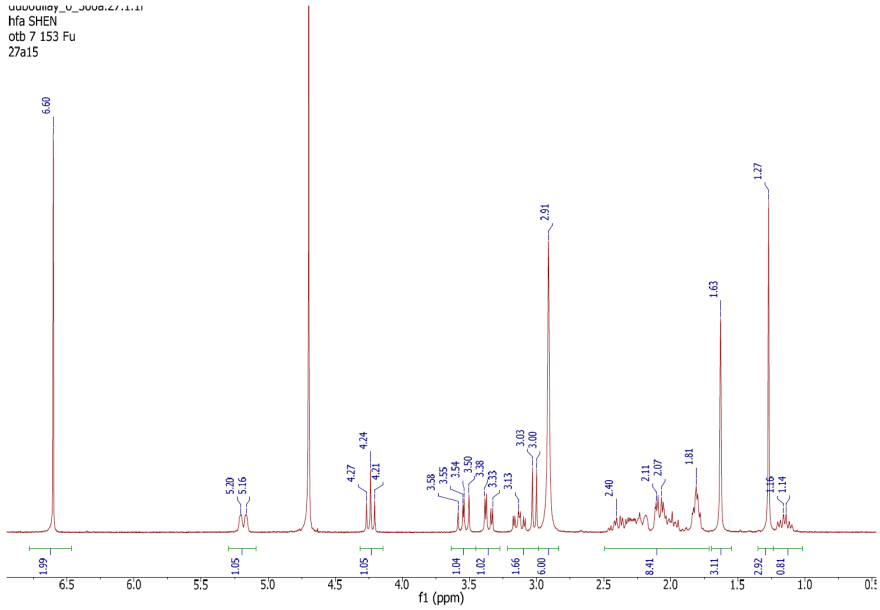

Figure S1C : DMAPT monofumarate, ^{1}H NMR (300 MHz, D_2O)

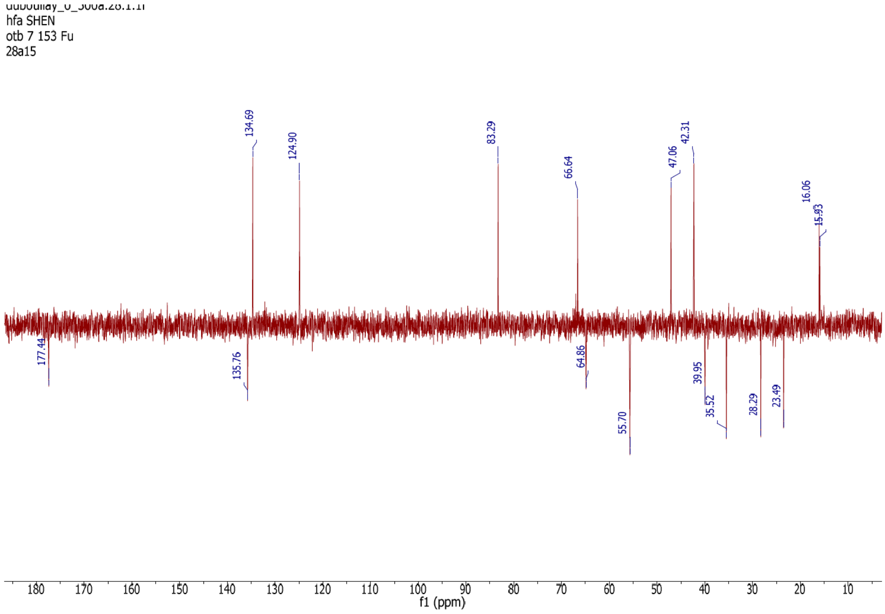

Figure S1D: DMAPT monofumarate, ^{13}C NMR (75 MHz, D_2O)

Figure S2: (A) Light microscopy of the suspension of glomeruli (arrows in A). Note the presence of numerous small shining spheres between the glomeruli that correspond to the Dynabeads. (B) Focuses on some glomeruli. Note the presence of the small black circles which correspond to the Dynabeads trapped by the glomeruli. (C) Expression of specific mRNA markers of glomeruli (Gl: nphs2, podxl and cldn5), proximal tubules (PT: aqp1, slc22a13), loop of Henlé (LHe: aqp1, slc12a3) and distal tubules (DT: wnk), in total kidney (white bars) and purified glomeruli (black bars). Values are means ± SEM of n=2.

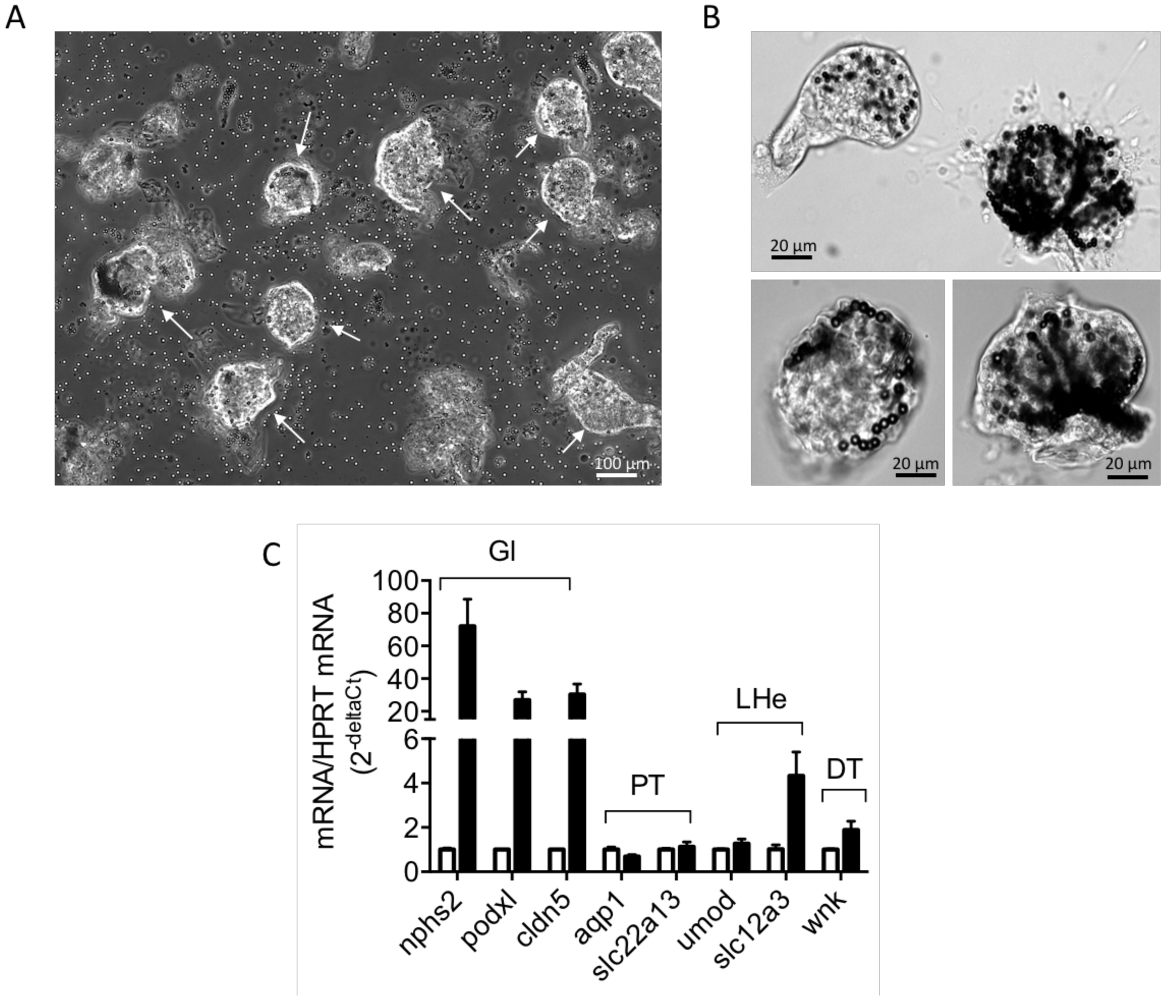

Figure S3: Kinetics of glycemia and ACR in Ins2 Akita mice. Blood and urine of wild type (black bars, n=20) and Ins2Akita (grey bars, n=19) mice were collected at different age, and glycemia (A) and ACR (B) were quantified. Values are mean ± SEM. *: P<0.05; **:P<0.01; ***: P<0.001; ****: P<0.0001 (unpaired t-test).

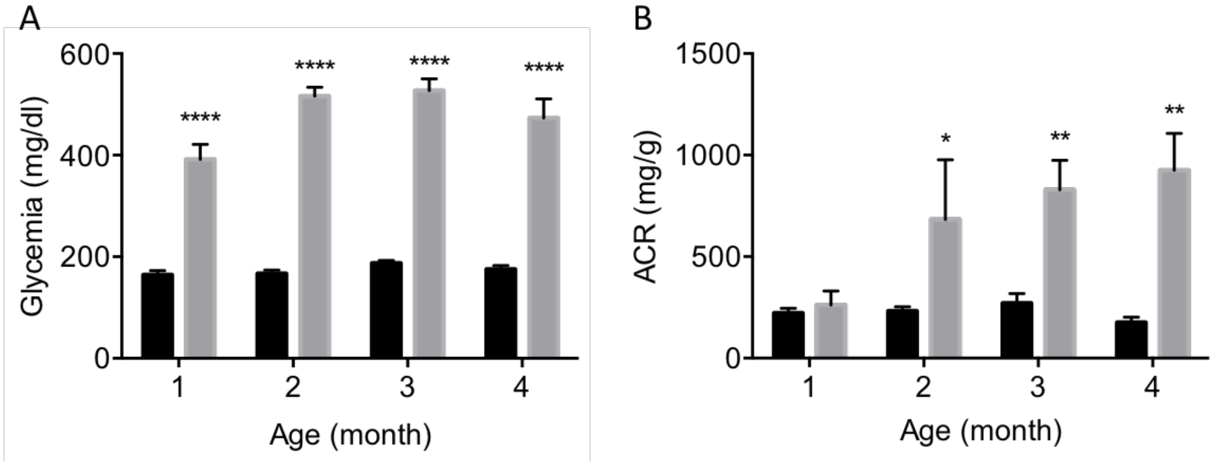

Figure S4: Flowchart of selection of ramipril-sensitive DKD-associated glomerular proteins (RS-DKD-GPs). Set#1 comparison led to the identification of 666 DKD-GPs out of which RI-DKD-GPs were selected according to their behavior in Set#2 and Set#3 comparisons. The figure drawn using Microsoft PowerPoint for Mac, Version 16.16.19).

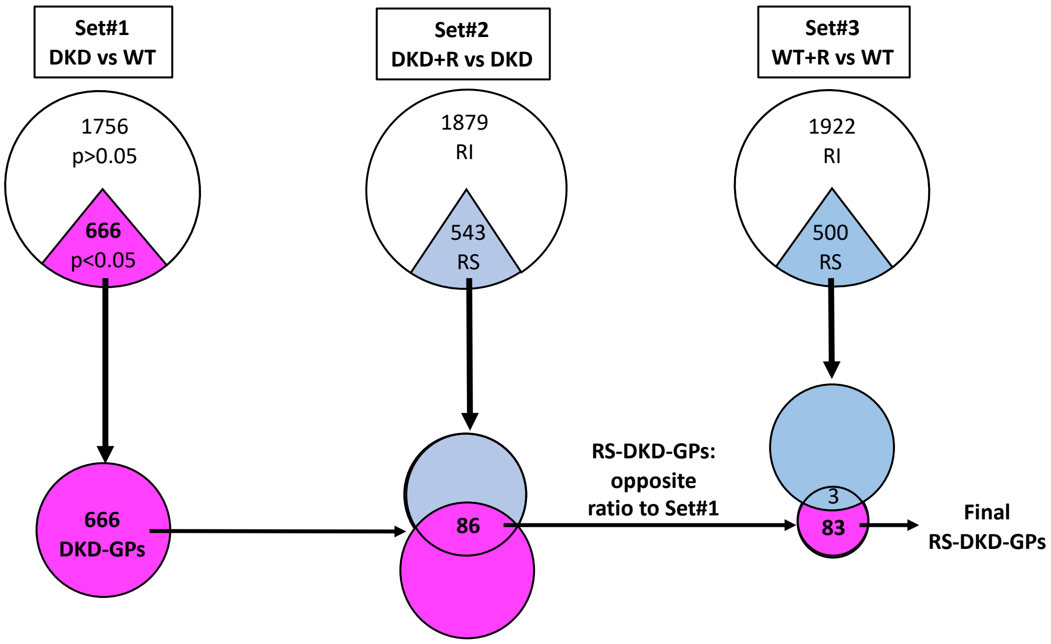

DKD-GPs: DKD-associated glomerular proteins
RI: ramipril-insensitive (p>0.05)
RS: ramipril-sensitive (p<0.05)
RI-DKD-GPs: ramipril insensitive DKD-associated glomerular proteins
