# Supplementary material for: Connectivity mapping of glomerular proteins identifies dimethylaminoparthenolide as a new inhibitor of diabetic kidney disease
Source: Sci Rep. 2020 Sep 10;10:14898. doi: 10.1038/s41598-020-71950-7 (PMC7484761; doi:10.1038/s41598-020-71950-7)
Supplement: Supplementary file 4 — Supplementary Legends. [file 41598_2020_71950_MOESM4_ESM.docx]

**Brief description of supplementary materials.**

### Protocols of DMAPT chemical synthesis.

### Figure S1 A-D. DMAPT purity evaluation by ^1^H and ^13^C NMR, mass spectrometry and melting point.

- **Figure S2.** (A) Light microscopy of the suspension of glomeruli (B) Focuses on some glomeruli. (C) Expression of specific mRNA markers of glomeruli, proximal tubules, loop of Henlé and distal tubules in total kidney and purified glomeruli.
- **Figure S3.** Kinetics of glycemia and urinary ACR in Ins2 Akita mice.
- **Figure S4.** Flowchart selection of ramipril-sensitive DKD-associated glomerular proteins (RS-DKD-GPs).
- **Table S1.**  NanoLC-MS/MS quantification of the glomerular proteins from WT, DKD and ramipril-treated DKD mice..
- **Table S2.** Significant up- and down-regulated DKD-GPs (Set#1) and their classification as RS- and Ri-DKD-GPs according to their ratio and p value in Set#2 abd Set#3.
